# Advanced Thyroid Follicular Carcinoma in a Pregnant Woman

**DOI:** 10.1155/2019/3064624

**Published:** 2019-12-28

**Authors:** Victor Rocha Pinheiro, Bruno Minoru Miamoto, Júlia Thalita Queiróz Rocha, Carlos Segundo Paiva Soares, José Vicente Tagliarini, Roberto Antônio de Araujo Costa, Glaucia Maria F. da Silva Mazeto, Mariangela Esther Alencar Marques, Cristiano Claudino Oliveira

**Affiliations:** ^1^Student of Medicine, Botucatu School of Medicine, São Paulo State University (FMB UNESP), Botucatu, São Paulo, Brazil; ^2^Pathologist and Postgraduate Student, Botucatu School of Medicine, São Paulo State University (FMB UNESP), Botucatu, São Paulo, Brazil; ^3^Otolaryngologist and Head and Neck Surgeon, Department of Ophthalmology, Otorhinolaryngology and Head and Neck Surgery, Botucatu School of Medicine, São Paulo State University (FMB UNESP), Botucatu, São Paulo, Brazil; ^4^Professor and Otolaryngologist and Head and Neck Surgeon, Department of Ophthalmology, Otorhinolaryngology and Head and Neck Surgery, Botucatu School of Medicine, São Paulo State University (FMB UNESP), Botucatu, São Paulo, Brazil; ^5^Professor and Obstetrician, Department of Obstetrician and Gynecology, Botucatu School of Medicine, São Paulo State University (FMB UNESP), Botucatu, São Paulo, Brazil; ^6^Professor and Endocrinologist, Department of Internal Medicine, Botucatu School of Medicine, São Paulo State University (FMB UNESP), Botucatu, São Paulo, Brazil; ^7^Professor and Pathologist, Department of Pathology, Botucatu School of Medicine, São Paulo State University (FMB UNESP), Botucatu, São Paulo, Brazil; ^8^Pathologist, Department of Pathology, Botucatu School of Medicine, São Paulo State University (FMB UNESP) and São Luiz/D'Or Hospital, São Paulo, Brazil

## Abstract

The diagnostic and therapeutic approach for pregnant women with thyroid nodules can present a challenge, especially concerning surgical procedures. In the context of malignant diagnosis, by fine needle aspiration (FNA), during pregnancy, the uncertainty lies in performing surgery. This article reports the case of a 41-year-old pregnant woman in her first gestation, who sought medical care complaining of right shoulder pain. Imaging workup depicted the destruction of the humeral head and involvement of the surrounding soft tissue. She was 20 weeks pregnant. The histological report favored the diagnosis of malignancy and the thyroid as the primary site. At 30 weeks of gestation, the patient underwent a cesarean section, a total thyroidectomy, and total resection of the metastasis. The child was born healthy, but one year after the diagnosis, the patient died. Bone and soft tissue metastasis of thyroid neoplasms are not very common and indicate poor prognosis.

## 1. Introduction

Thyroid nodules are common entities, and malignancy is found in 5% to 10% of them [1]. The finding of a thyroid nodule in a cervical ultrasonographic examination, depending on the size and radiological characteristics standardized by the Thyroid Imaging Reporting and Data System (TIRADS), may require further investigation with fine needle aspiration (FNA) and, possible surgery depending on the cytological findings. This scenario can be challenging to manage during pregnancy [1, 2]. This article presents the clinical and pathological features of advanced-stage thyroid carcinoma diagnosed in a pregnant woman.

## 2. Case Presentation

A 41-year-old pregnant woman, in her first gestation, sought medical care complaining of pain in her right shoulder for the last two months, associated with the impairment of moving her arm. She was 20 weeks pregnant and did not attend any prenatal consultation. Her medical history included smoking, correctly treated syphilis, and pulmonary thromboembolism. The patient did not have any family history of thyroid disease.

**Figure 1:**
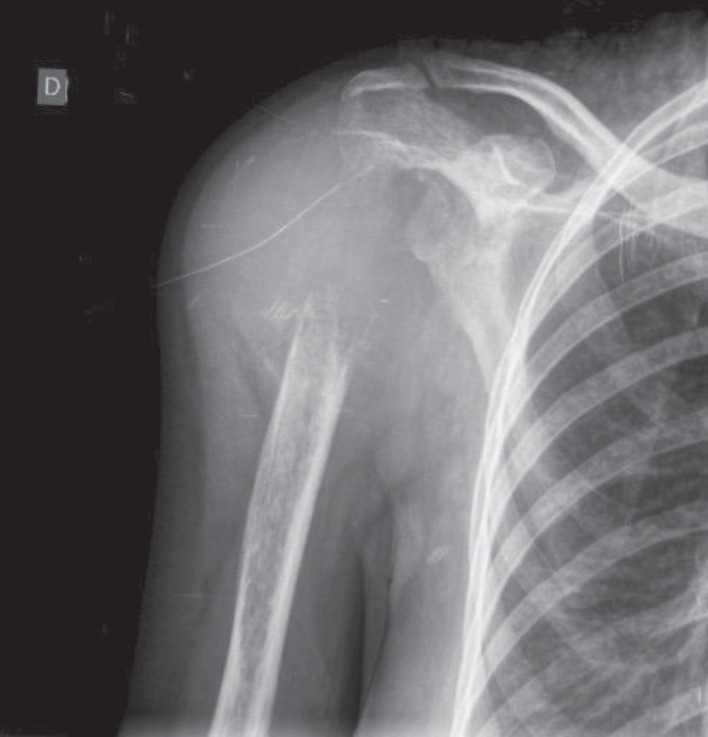
Right arm radiographic. There is a lytic bone lesion on her shoulder with infiltration of local soft tissue. Core biopsies were performed from this site.

**Figure 2:**
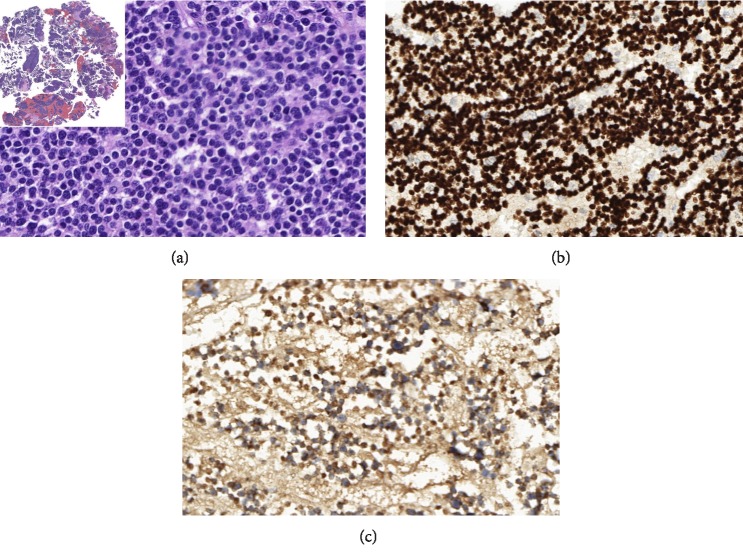
(a) (H&E, 400x and 20x). Right arm lesion biopsy. There are two images. The small one is a panoramic view with many fragments of the sample. The large one, with a high magnification, demonstrates the follicular aspect of the atypical cells. (b) (400x, TTF1). The neoplastic cells are positive for TTF1, indicating thyroid as a possibility of primary site for this case. (c) (400x, thyroglobulin). Besides the positivity for TTF1, the thyroglobulin pattern of positivity indicates thyroid as primary site of the cancer.

Right arm radiography (Figure 1) demonstrated a lytic bone lesion on her shoulder, apparently involving the surrounding soft tissues. The initial working diagnosis was a soft tissue or bone tumor with differential secondary lesions, for example, metastatic carcinomas. The patient was submitted to a biopsy of the lesion (Figure 2), which revealed a high-grade malignant neoplasm with an epithelioid aspect on the histological examination. The predominant pattern was solid with sheets of atypical cells and a sparse microfollicular-like arrangement. There were many mitoses and, occasionally, nuclear hyperchromasia. The morphological hypothesis was a metastatic carcinoma and, more remotely, primitive neuroectodermal neoplasms.

The immunohistochemistry (IHC) was diffuse and strongly positive for cytokeratin (AE1/AE3) and TTF1. CD45, CD99, chromogranin-A, and synaptophysin were negative. Since the morphology was favorable for thyroid differentiation, the IHC panel was extended to thyroglobulin, calcitonin, and surfactant with a positive result only for thyroglobulin. Thus, the diagnosis of metastatic thyroid carcinoma was made. Also, the thoracic computerized tomography (CT) failed to show any pulmonary lesion. The thyroid examination revealed an increased cervical volume, with some firm and painful areas, in the paramedian region. It is important to mention that until that moment the patient had no previous medical care. The cervical enlargement observed in the anterior portion of the neck was not reported by the patient. When asked, the patient reported that there might have been some increase in the region.

**Figure 3:**
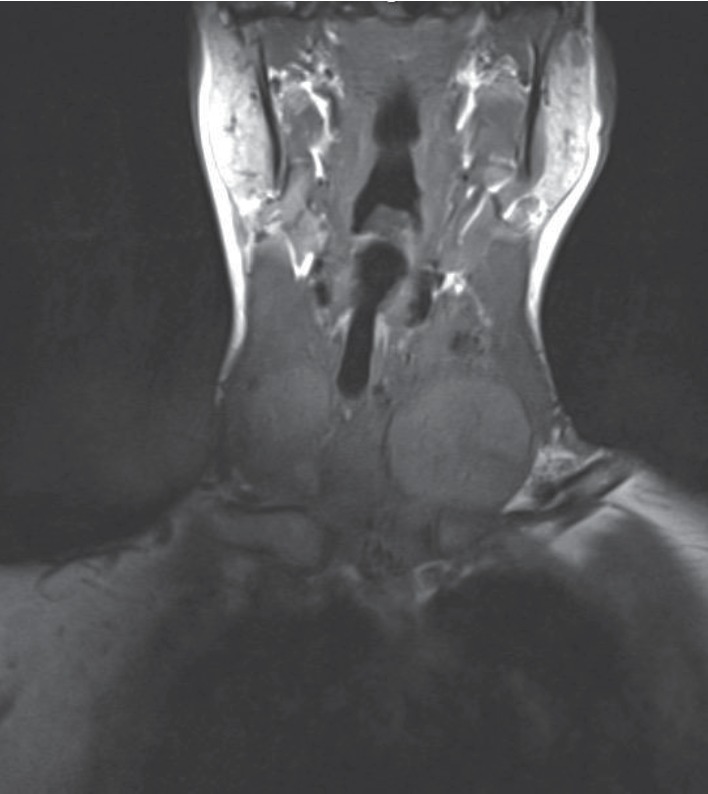
Nuclear magnetic resonance (NMR) revealed a suspected nodule localized in left lobe of thyroid, measuring approximately 4.0 cm in diameter. Besides this, the thyroid increased in size and, clinically, the lesion may extend from the left for the right side.

**Figure 4:**
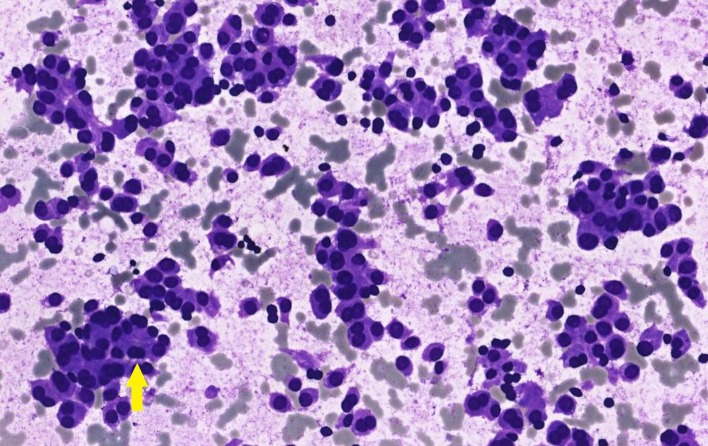
(400x, Giemsa). FNA of thyroid nodule. There is a hypercellular smear with atypical cells and small follicle indicated by a yellow arrow. Other cells have a oncocytic aspect.

The ultrasonography (US) and magnetic resonance imaging (MRI) revealed a suspected nodule localized in the left lobe of the thyroid, measuring 4.0 cm in diameter (Figure 3). An FNA was performed, and cytological evaluation was consistent with category IV of Bethesda Classification, suspected for follicular neoplasm (Figure 4).

**Figure 5:**
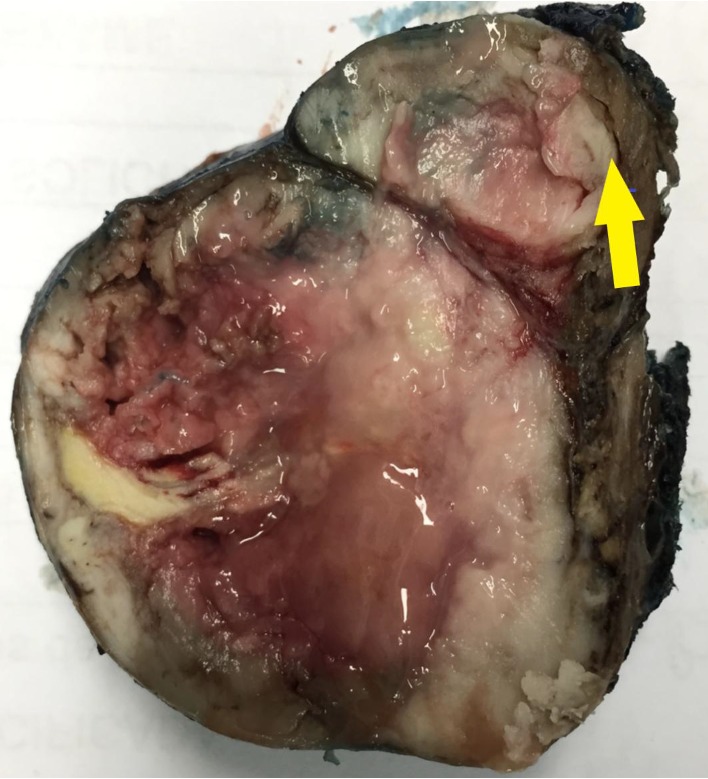
Thyroid, marcocopy image. The tumor measured 4.0 × 4.0 × 3.0 cm, in the left thyroid lobe. It was an expansive tumor with macroscopic infiltration of the capsule, as demonstrated by the arrow.

After this diagnosis, the option for treatment was a total thyroidectomy. The macroscopic examination demonstrated a diffusely increased glandule where there was a nodule measuring 4.0 × 4.0 × 3.0 cm in the left thyroid lobe. It was an expansive tumor with macroscopic infiltration of the capsule (Figure 5).

**Figure 6:**
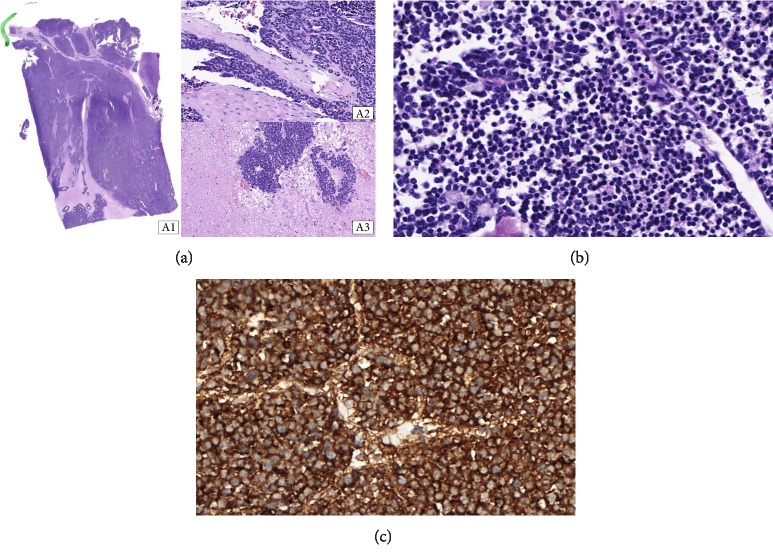
(a) (20x, H&E). A1—A tumor image which demonstrated the aggressiveness of the neoplasm. There is an expansive pattern of growing and the area of capsule infiltration. This area is the upper part of the figure. A2—Vascular infiltration by the tumor cells. A3—Necrosis and neoplasm. (b) (400x, H&E). The neoplasm has nuclear pattern of follicular carcinoma. There are no nuclear grooves or pseudo inclusions. Papillary differentiation was declined. (c) (400x, thyroglobulin). The lesion is positive in a diffuse pattern for thyroglobulin which confirms the thyroid carcinoma.

The histopathologic finding was similar to the bone/soft tissue lesion biopsy. Immunohistochemistry results were the same (Figure 6). The final diagnosis was follicular carcinoma of the thyroid, with follicular (90% of the tumor) and solid (10% of the tumor) patterns accompanied by necrosis, vascular infiltration (at least six areas), and lymphatic infiltration. Mitosis number is two in ten high magnification microscopic fields. Near the thyroid, there were two lymph nodes without neoplasm. However, no specific resection of cervical lymph nodes was performed. The pathological stage was pT3a pN0 pM1 (AJCC, 2017). The tests performed were: IHC markers such as thyroglobulin and TTF1 with a diffuse pattern of immunostaining. The Ki-67 index was compatible with the mitosis number (8%), and the p53 marker was negative.

A cesarean was performed at the same time, at 30 weeks of pregnancy. After three months, the patient underwent surgery of the right arm to install a prosthetic. She received therapeutic iodine treatment and had medullary compression syndrome, secondary to the bone and spinal involvement. There were no other sites of metastasis. The disease progressed, and the patient died one year after the diagnosis. The child survived without morbidities.

## 3. Discussion

Thyroid malignancies rarely develop bone metastasis, occurring in approximately 4.0% of patients. Medullary and follicular carcinomas of the thyroid are the types most related to metastasis. In general, bone lesions in the context of thyroid cancer are associated with a worse prognosis than other metastatic sites [3].

The diagnosis was a follicular thyroid carcinoma, but a discussion about the differential with poorly differentiated thyroid carcinoma (PDTC) is very relevant. The PDTC is a follicular neoplasm with limited evidence of follicular cell differentiation whose diagnosis is defined based on Turin proposal. The PDTC histopathology is a high-grade carcinoma with solid pattern and focal areas of microfollicular growth, without nuclear features of papillary carcinoma. In addition, in the IHC, these tumors have decreased expression of thyroglobulin. In the case presented in this article, the neoplasm pattern was follicular in 90% of the tumor, and only 10% was solid. Mitosis number and proliferation index were low. The thyroglobulin was diffused in the neoplasm. Thus, in this context, the diagnosis was a tumor of the follicular carcinoma. However, it is important to show that the Turin consensus explains that the PDTC can be pure or with coexistent follicular carcinoma. But, in those cases, the follicular areas are smaller than the poorly differentiated areas. The patient reported here had a follicular carcinoma with a solid pattern area, without a cytological high grade. The behavior was aggressive, and, interestingly, the metastasis lesion had a higher grade than the thyroid lesion.

Human chorionic gonadotropin (hCG) share a common alpha subunit with thyrotropin (TSH). During pregnancy, hCG, and TSH work as thyroid-stimulators, which can favor the growth, progression, and spread of thyroid tumors, mainly papillary carcinomas [4].

The American Thyroid Association (ATA) recommends ultrasonography for evaluation of thyroid nodules, including during pregnancy. The ATA suggests monitoring the growth of malignant nodules during pregnancy, and if the nodule grows, the pregnancy should be interrupted after 24 weeks. The American Endocrine Society's guidelines state that FNA should be performed for thyroid nodules with solid pattern and larger than 1.0 cm in diameter during the pregnancy [4].

Cancer in a pregnant woman is a clinical challenge. In the case of thyroid, the objective is to postpone the oncological surgery. Some authors suggested the use of TSH suppression for postponing the thyroid disease managing it until the postpartum period [4].

In a study [4] comprising of 50 women with micropapillary carcinoma of the thyroid observed during their pregnancy, 4 (8%) patients had an increase in the tumor size, and the remaining had unchanged nodules during the pregnancy. None of them had metastasis. Although this sample is small, these numbers reflect the idea that the tumor may progress, probably due to the hormonal influence. According to Durante et al. [5], approximately 20% of patients with treated thyroid papillary carcinoma had recurrence many years after the treatment. There is no evidence that pregnancy is a risk factor for the development of thyroid cancer. Hirsh et al. [6] did not find any correlation of disease progression with TSH levels before and during pregnancy or with the interval between the first diagnosis of malignancy and the pregnancy.

There is no consensus on the timing for the surgical treatment of pregnant women with thyroid malignancy. The available evidence is based on nonrandomized retrospective studies, but they indicate that the majority of well-differentiated thyroid malignancies in pregnancy have slow growth and good prognosis. Thus, oncological treatment may be delayed until after delivery. However, the case reported here involves a diagnosis of advanced and metastatic disease [7].

Treatment for metastatic disease in pregnancy is challenging. It holds risks for both mother and child, which are essential points to be considered by clinicians and the family, in a multidisciplinary team, if it is possible [8]. According to the different authors discussed in this article, pregnancy is not a risk factor for the neoplasm or progression of the disease. Pregnancy does not seem to cause direct changes in the prognosis. However, metastatic disease is a relevant marker of poor outcome [8, 9], as occurred with this patient, independent of her gestational condition.

## References

[1] Rosas S. (2003). Thyroid Nodule and Pregnancy. *Acta Médica Portuguesa*.

[2] Tuttle R. M., Morris L. F., Haugen B. R. (2017). Thyroid—Differentiated and Anaplastic Carcinoma. *American Joint Committee on Cancer. AJCC Cancer Staging Manual*.

[3] Choksi P., Papaleontiou M., Guo C., Worden F., Banerjee M. (2017). Megan haymart; skeletal complications and mortality in thyroid cancer: a population-based study. *The Journal of Clinical Endocrinology & Metabolism*.

[4] Ito Y., Miyauchi A., Kudo T. (2016). Effects of pregnancy on papillary microcarcinomas of the thyroid re-evaluated in the entire patient. *Thyroid*.

[5] Durante C., Montesano T., Torlontano M. (2013). Papillary thyroid cancer: time course of recurrence during postsurgery surveillance. *The Journal of Clinical Endocrinology & Metabolism*.

[6] Hirsch D., Levy S., Tsvetov G. (2010). Impact of pregnancy on outcome and prognosis of survivors of papillary thyroid cancer. *Thyroid*.

[7] Modesti C., Aceto P., Masini L., Lombardi C. P., Bellantone R., Sollazzi L. (2017). Approach to thyroid carcinoma in pregnancy. *Updates in Surgery*.

[8] Rowe C. W., Murray K., Woods A., Gupta S., Smith R., Wynne K. (2016). Management of metastatic thyroid cancer in pregnancy: risk and uncertainty. *Endocrinology, Diabetes & Metabolism Case Reports*.

[9] Yu S. S., Bischoff L. A. (2016). Thyroid cancer in pregnancy. *Seminars in Reproductive Medicine*.

